# Leukocyte activation and inflammation in acute myocardial infarction: novel insights from cell population data

**DOI:** 10.3389/fimmu.2026.1864687

**Published:** 2026-07-03

**Authors:** Damien Leleu, Maxime Nguyen, Julien Guy, Michel Farnier, Frédéric Chagué, Laurine Collas, Margot Machado, Marine Gougeon, Jean-Paul Pais de Barros, Florence Bichat, Maud Maza, Yves Cottin, David Masson, Marianne Zeller

**Affiliations:** 1 Université Bourgogne Europe, CHU Dijon Bourgogne, Laboratory of Clinical Chemistry, INSERM, CTM UMR1231, LIPNESS, Dijon, France; 2 LipSTIC LabEx, Dijon, France; 3 Université Bourgogne Europe, CHU Dijon Bourgogne, Department of Anaesthesiology and Intensive Care, INSERM, CTM UMR1231, LIPNESS, Dijon, France; 4 Université Bourgogne Europe, CHU Dijon Bourgogne, Laboratory of Clinical Haematology, Dijon, France; 5 Université Bourgogne Europe, Physiopathologie et Epidémiologie Cérébro-Cardiovasculaire (PEC2), Dijon, France; 6 Université Bourgogne Europe, CHU Dijon Bourgogne, Cardiology Department, Dijon, France

**Keywords:** CPD, immature granulocyte, inflammation, monocyte, myocardial infarction, neutrophil

## Abstract

**Background:**

Immune dysregulation is strongly involved in acute myocardial infarction (AMI), driving sustained inflammation and myocardial damage. A better understanding of the immune cell response to AMI is needed, beyond neutrophil and monocyte counts. Cell Population Data (CPD) analysis, including granularity (SSC), size (FSC), activation (SFL), and heterogeneity indices, offers deeper insights into the dynamics of leukocyte populations.

**Objective:**

Our goal was to investigate CPD as prognostic biomarkers for cardiovascular (CV) mortality after AMI and to assess their links to inflammatory and lipid markers.

**Methods:**

Samples from 572 patients with AMI were collected at the admission in the cardiology intensive care unit. In addition to routine blood tests, CPD was acquired on an automated haematology analyser. Plasma inflammatory markers, as well as detailed fatty acid profiles, were also measured. A one-year follow-up was then conducted to assess CV mortality.

**Results:**

Neutrophil, monocyte, and immature granulocyte (IG) counts and neutrophil heterogeneity of fluorescence (NE-WY) were associated with CV deaths, myocardial infarction (MI), and inflammatory biomarkers such as hs-CRP and IL-6. On the opposite, lymphocyte count was inversely associated with CV death and inflammatory markers. Monocyte count and CPD were correlated with saturated fatty acids, especially palmitic acid.

**Conclusion:**

Our findings demonstrate that CPD, particularly parameters related to neutrophils and monocytes, are robustly linked to inflammation and the occurrence of CV mortality. Importantly, these CPD—readily accessible through standard clinical haematology analysers—hold significant potential as predictive biomarkers for clinicians to assess cardiovascular risk in routine practice.

## Graphical Abstract

**Figure:**
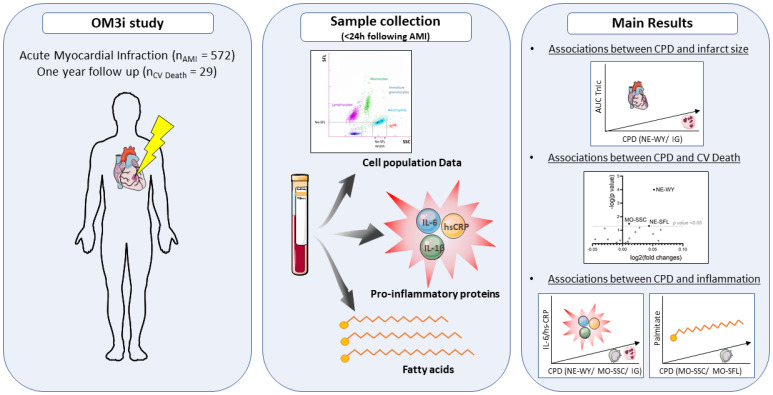

## Introduction

Over the last decades, dysregulation of the immune and inflammatory systems has emerged as a potential therapeutic target in patients with acute myocardial infarction (AMI) (1, 2). The inflammatory response to AMI is complex, involving several immune cell types, with dynamic and divergent roles in the early phase. Among all leukocytes involved, neutrophils are now recognized as crucial contributors to cardiovascular diseases (CVD), with elevated neutrophil counts strongly linked to worse prognosis across a wide range of CVD, including atherosclerosis, MI, heart failure, and peripheral artery disease (3, 4). Neutrophils contribute to the progression of CV lesions through multiple mechanisms, including the release of proteolytic enzymes, production of reactive oxygen species, and formation of neutrophil extracellular traps (NETs) (3). Altogether, these processes promote vascular inflammation, thrombosis, and tissue degradation. Several large cohort studies have shown that high neutrophil counts are associated with a higher risk of CV events, including CV mortality (5–7). The neutrophil-to-lymphocyte ratio (NLR) is a particularly robust predictor, outperforming individual cell counts in prognostic accuracy for coronary heart disease and hypertension (5, 6). In parallel, growing evidence suggests that plasma lipid composition, and in particular fatty acid profiles, may modulate neutrophil activation and systemic inflammation, thereby influencing cardiovascular outcomes (8, 9).

In this context, routine blood cell counts represent an accessible source of information to estimate CV risk in patients with AMI. Most automated haematology analysers quantify blood cell populations using flow cytometry methods, which may provide other information, beyond standard data on clinical reports. These Cell Population Data (CPD) include leukocyte characteristics such as granularity or internal complexity (side scatter SSC), size (forward scatter FSC), fluorescence (side fluorescence SFL), which is associated with cell activation or nucleic acid content. Moreover, side heterogeneity indices (WX, WY, and WZ) offer insight into the diversity of cell states in inflammation or recovery phases (10). CPD have demonstrated significant diagnostic and prognostic value in many diseases, especially in sepsis, infections, haematological malignancies, and cancer (11). Small-sized retrospective studies have shown that immature granulocytes (IG) levels were associated with early mortality (12, 13). Moreover, there is only limited data on the association between neutrophil fluorescence and CVD (10).

This study aimed was to determine whether CPD can be used as a prognostic biomarkers for CV mortality in patients with AMI. We hypothesized that analysis of CPD at admission, particularly neutrophil-related parameters, would identify inflammatory profiles associated with an increased death rate at the 1year follow-up. In addition to clinical outcomes, we also sought to explore the relationship between CPD and key inflammatory and lipid markers, including detailed fatty acid (FA) profiles, to better characterize the biological pathways connecting neutrophil activation, systemic inflammation, and cardiovascular risk. To address this, we retrospectively analysed clinical and biological data from AMI patients recruited in the OM3i study at the cardiology department of the Dijon University Hospital (14, 15). For each patient, CPD measurements were acquired upon admission using automated haematology analysers and were compared with clinical and biological data, as well as with the occurrence of CV death at the one-year follow-up. Among the CPD, neutrophil-related indices emerged as the most informative, showing the strongest and significant associations with inflammatory parameters and clinical outcomes.

## Patients and methods

### Protocol

This work is an ancillary study of the OM3i (Relation between OMega-3 fatty acids and Inflammation in patients with acute myocardial infarction) cohort described previously (14, 15). Briefly, from January 1, 2021, to May 30, 2022, consecutive patients admitted <24 h after symptom onset to the coronary care unit of the Dijon University Hospital for type 1 MI (atherosclerotic plaque rupture) were included. AMI was diagnosed by an increase in serum cardiac troponin I (TnIc) associated with symptoms of ischemia and/or typical electrocardiogram signs in agreement with the universal definition of MI. Patients with chronic inflammatory diseases or on anti-inflammatory drugs were excluded. Participants provided written informed consent before inclusion and the investigation conformed to the principles outlined in the Declaration of Helsinki. The ethics committee approved the protocol registered on health-data-hub (https://health-data-hub.fr, #F20210727150424). All characteristics of the patients at admission, during the hospital stay and at the one-year follow-up were previously described (14, 15).

### Sample processing

The method has been described previously (14). Briefly, heparinized venous blood samples were harvested upon admission and centrifuged within 1 h after collection. Biological parameters were analyzed on an Atellica^®^ analyzer (Siemens Healthcare^®^). Creatinine, total cholesterol, HDL-cholesterol, triglycerides, and glucose were measured by spectrophotometry, NT-ProBNP, TnIc were measured by chemiluminescence, and high sensitive C-reactive protein (hs-CRP) was measured by a latex-enhanced immunoturbidimetric assay. LDL-Cholesterol was calculated using the Friedewald formula or measured if triglyceride concentration was >350 mg/dL. Estimation of glomerular filtration rate (eGFR) was calculated according to Chronic Kidney Disease EPIdemiology collaboration (CKD-EPI) equation (16). Plasma was collected in Lithium Heparin tubes and stored at -80 °C until cytokine assays were performed.

### Area under the curve of troponin Ic

Area under the curve (AUC) of TnIc was used as an estimation of infarct size (17, 18). All TnIc concentrations, measured in patient samples collected within 96 hours following the admission, were collected. Then, AUC of TnIc concentrations was calculated for each patient using the trapezoidal method, which approximates the AUC by summing the areas of successive trapezoids formed between each pair of consecutive time points. This approach allows for an accurate estimation of total TnIc exposure over the 96-hour observation period (**Supplementary Figure 1**). All calculations were performed using Python (version 3.11) with the NumPy library, ensuring precision and reproducibility of the results.

### CPD analysis

For each patient, a complete blood cell count was performed on automated hematology analyzer (Sysmex XN-9000, Sysmex, France). As previously described (10), this analyser was equipped with impedance and fluorescence flow cytometry devices, which generate detailed CPD. These parameters include morphological indices such as FSC, which reflects cell size, and SSC, which indicates internal cell structure and granularity. Activation and metabolic activity are assessed through SFL, which reflects DNA/RNA content. Finally, heterogeneity within cell populations is captured by WX (heterogeneity in cell complexity), WY (heterogeneity in fluorescence intensity), and WZ (heterogeneity in cell size). With these characteristics, different leukocyte populations can be identified (Neutrophils, Lymphocytes, Monocytes, and IG) (**Supplementary Figure 2**). For each population, cell count (expressed as ×10³ cells/µL) and CPD (expressed as mean fluorescence intensity [MFI]) were retrospectively extracted.

### Protein quantification

Concentrations of interleukin-6 (IL-6) and myeloperoxidase (MPO) were measured by enzyme-linked immunosorbent assay (ELISA) (Human IL-6 DuoSet ELISA, Catalog # DY206, and Human Myeloperoxidase DuoSet ELISA, Catalog # DY3174, Biotechne, respectively) according to the manufacturer’s instructions, with a limit of quantification (LOQ), the lowest quantifiable concentration on the calibration curve. Plasma interleukin-1β (IL-1β) concentration was determined using a high-sensitivity ELISA (Human IL-1b High Sensitivity ELISA Kit, Catalog # BMS224-2HS, ThermoFisher Scientific) with a limit of quantification (LOQ) of 0.05 pg/mL. For each of them, internal quality control measures showed inter-assay coefficient of variation below 15%.

### Fatty acid quantification

The lipidomic method has been described previously (14). Briefly, total FAs were quantified and analysed as penta-fluorobenzyl esters by gas chromatography-mass spectrometry (Thermo-Electron Corp., Waltham, USA). Gas chromatography-mass spectrometry analysis was carried out on a 7890A GC system equipped with a 7683 injector and a 5975C mass selective detector (Agilent Technologies). For each patient, the results for each FA species were then analysed as a proportion of total FA.

### Endpoints

The primary endpoint was the occurrence of CV death during the first year after inclusion. Secondary endpoints included myocardial necrosis size, reflected by the AUC of TnIc, and inflammation, using the following parameters: IL-1β, IL-6, MPO, hs-CRP and FA.

### Statistical analysis

Dichotomous variables were expressed as n (%) and continuous variables as median (interquartile range). The Kolmogorov–Smirnov test was applied to assess the normality of variables. None of them were normally distributed; thus, the Mann−Whitney test was used to compare continuous data, and correlation analyses were performed using the Spearman’s method. One-year survival was represented using Kaplan–Meier curves (CV mortality) and compared with the log-rank test. The optimal threshold for discriminating CV mortality was determined using Youden’s index from the Receiver Operating Characteristic (ROC) curve. A multiple logistic regression model was used to estimate CV mortality. The multivariate models were built by including predictive variables in univariate analysis, based on their clinical relevance, and an inclusion threshold of p<0.01. Quality indices of the models, as assessed by LogLikelihood (LL) test, were satisfactory. Statistical tests were two-tailed and a p<0.05 was considered significant. The Benjamini-Hochberg correction was applied for multiple comparisons. Missing data were at random and were omitted. GraphPad Prism 8 was used for all the tests and for creating the figures.

## Results

### Baseline characteristics

Among the 572 patients included, 43 deaths occurred within the one-year follow-up, including 9 in-hospital deaths, 29 of which were of CV origin (**Supplementary Figure 3**). Patient characteristics at admission, along with the comparison between living and deceased patients, have been previously described (15) and were reported in **Supplementary Table S1**. When compared with living patients, patients with CV death were older (76 (67–86) vs. 67 (57–76) y, p=0.002), had a lower BMI (24 (23–29) vs. 27 (24–30) kg/m², p=0.01), a higher prevalence of coronary artery disease (CAD) history (38 vs. 19%, p=0.016), lower eGFR (58 (39–90) vs. 89 (71–99) mL/min/1.73m², p<0.001), had a weaker left ventricular ejection fraction (LVEF)(34 (26.5-50.5) vs. 55 (45–60)%, p<0.0001), and had higher levels of inflammatory biomarkers such as high-sensitivity C-reactive protein (hs-CRP) (11.4 (4.0-67.6) vs. 3.4 (1.2-10.0) mg/L, p<0.001).

### Admission CPD are associated with 1 year cardio-vascular mortality and myocardial injury

**Figure 1:**
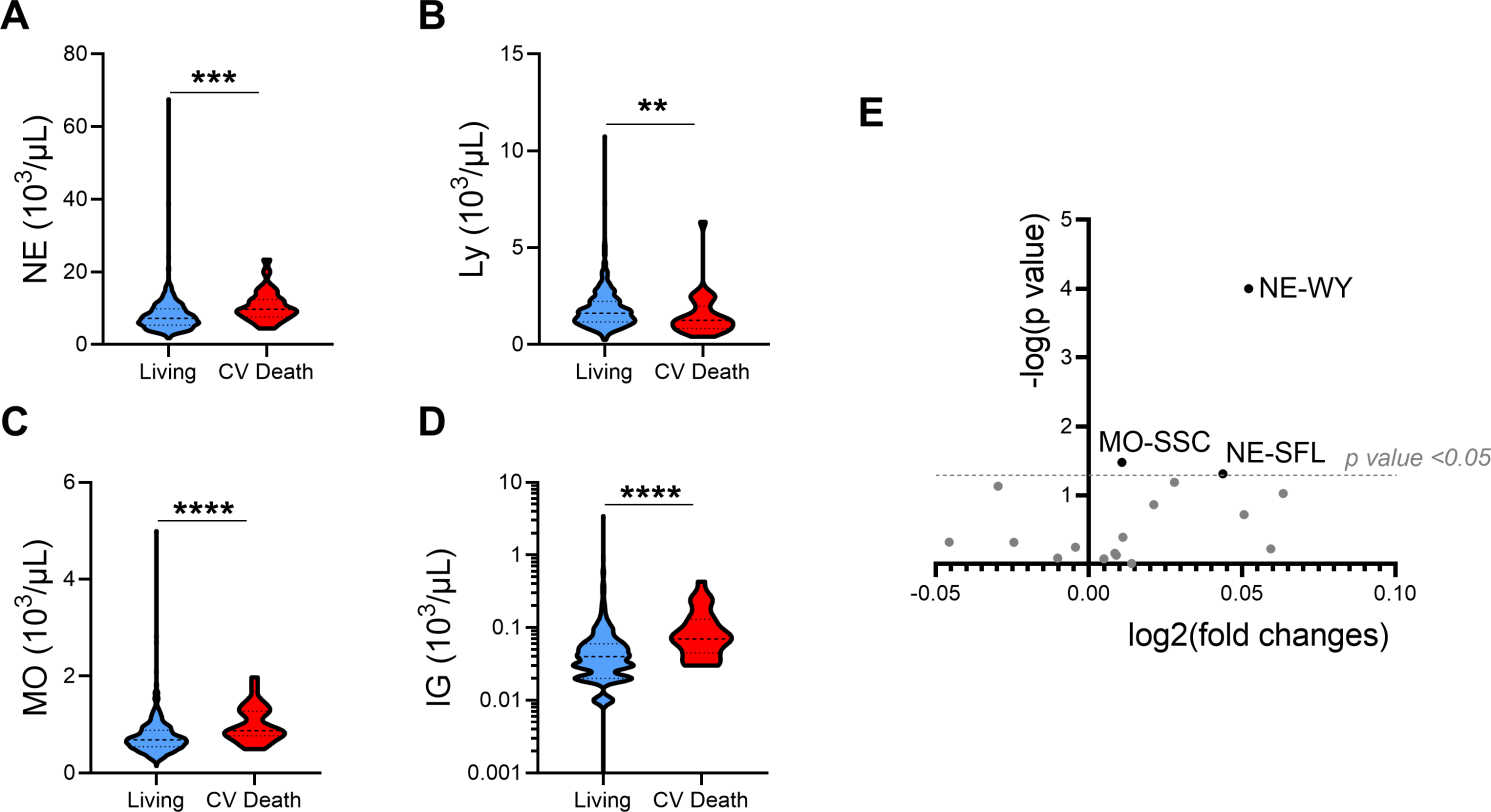
Comparison of patient characteristics and cell population data in relation to one-year mortality. **(A-D)** Comparison of cellular concentrations of neutrophils **(A)**, lymphocytes **(B)**, monocytes **(C)**, and immature granulocytes **(D)** between living patients (n=529) and those who died from cardiovascular causes within one year of inclusion (n=29). **(E)** Volcano plot illustrating differential cell population data between deceased (n=29) and living subjects (n=529). X-axis: Log_2_ fold change (cardiovascular deceased/living), Y-axis: −log_10_(p-value). Dashed line represents a p-value of 0.05. **p<0.01, ***p<0.001, ****p<0.0001 Mann Whitney test was performed for all tests. CV, cardiovascular; NE, Neutrophils; LY, Lymphocytes; MO, Monocytes; IG, Immature Granulocytes; SFL, side fluorescence light intensity; WY, heterogeneity in fluorescence intensity; SSC, side-scattered.

Regarding the leukocyte counts, when comparing living patients with those who died within the first year from a CV cause, deaths exhibited higher concentrations of neutrophils, monocytes, and IGs (neutrophils: 9.69 [7.81 - 12.33] vs. 7.21 [5.39 - 9.73] x10³/µL, p=0.0002; monocytes: 0.87 [0.77 - 1.27] vs. 0.68 [0.54 - 0.88] x10³/µL, p<0.0001; IG: 0.04 [0.02 - 0.06] vs. 0.07 [0.04 - 0.13] x10³/µL, respectively, p<0.0001; **Figures 1A, C, D**). On the other hand, lymphocyte concentration was significantly lower in patients who died (1.26 [0.84 - 1.88] vs. 1.62 [1.17 - 2.23] x10³/µL, p=0.010, **Figure 1B**). For CPD, comparisons between living and CV-related death patients are shown in **Figure 1E** as a volcano plot. Among these parameters, only NE-SFL, NE-WY and MO-SSC were significantly increased in the CV death group (NE-SFL: 46.90 [45.5 - 48.3] vs. 45.50 [43.8 - 47.5] MFI, p=0.0487; NE-WY: 648.0 [638.0 - 680.0] vs. 625.0 [604.0 - 650.5] MFI, p<0.0001; MO-SSC: 120.60 [119.0 - 122.5] vs 119.70 [118.0 - 121.5] MFI, p=0.0334; **Figure 1E**). These initial results suggest a potential link between neutrophil profile and CV mortality.

Associations between CPD and infarct size, as estimated by the AUC of TnIc concentrations, are presented in **Table 1**. Among neutrophil data, positive correlations were observed between TnIc AUC and cell count, SSC, SFL, FSC, and NE-WY. For lymphocytes, a negative correlation was detected between TnIc AUC and SFL. Within the monocyte population, TnIc AUC was negatively correlated with SSC and SFL, but positively correlated with FSC. IG concentration exhibited a strong positive correlation with TnIc AUC. These results suggest that among the different cell populations, neutrophil CPD, in particular NE-WY had the strongest association with myocardial injury.

**Table 1:** Characteristics of cell population data according to troponin Ic area under the curve [TnIc AUC (ng/L/h)] as a reflect of infarct size.

TnIc AUC (ng/L/h)		
	r	P-value
Polymorphonuclear neutrophils		
Concentration (103/µL)	0.386	<0.0001
SSC (MFI)	0.163	<0.0001
SFL (MFI)	0.090	0.0317
FSC (MFI)	0.214	<0.0001
NE-WX (MFI)	-0.044	0.2980
NE-WY (MFI)	0.201	<0.0001
NE-WZ (MFI)	0.021	0.6131
Lymphocytes		
Concentration (103/µL)	-0.077	0.0669
SSC (MFI)	0.022	0.5948
SFL (MFI)	-0.139	0.0008
FSC (MFI)	0.009	0.8241
LY-WX (MFI)	-0.009	0.8126
LY-WY (MFI)	-0.053	0.205
LY-WZ (MFI)	0.021	0.6209
Monocytes		
Concentration (103/µL)	0.061	0.1451
SSC (MFI)	-0.092	0.0285
SFL (MFI)	-0.119	0.0043
FSC (MFI)	0.127	0.0023
MO-WX (MFI)	-0.021	0.6209
MO-WY (MFI)	0.032	0.4413
MO-WZ (MFI)	-0.011	0.7917
Immature granulocytes		
Concentration (10^3^/µL)	0.255	<0.0001

Table showing the associations between CPD and TnIc AUC for 572 patients. Spearman correlation test was performed.

AUC, Area Under the Curve; SSC, side scatter; FSC, forward scatter; SFL, side fluorescence light; NE-WX, neutrophil heterogeneity in cell complexity; NE-WY, neutrophil heterogeneity in fluorescence intensity; NE-WZ, neutrophil heterogeneity in size; LY-WX, Lymphocytes heterogeneity in cell complexity; LY-WY, Lymphocytes heterogeneity in fluorescence intensity; LY-WZ, Lymphocytes heterogeneity in size; MO-WX, Monocytes heterogeneity in cell complexity; MO-WY, Monocytes heterogeneity in fluorescence intensity; MO-WZ, Monocytes heterogeneity in size; r, correlation coefficient.

We further investigated the clinical and biological determinants of these parameters of interest (NE-WY, MO-SSC, and IG) (**Supplementary Tables 2–4**). NE-WY was positively associated with age, diabetes, haemoglobin A1c (HbA1c), and clinical heart failure (as defined by Killip class > 2) and inversely associated with LVEF and eGFR. MO-SSC was positively associated with age, diabetes, HbA1c, and CAD and inversely associated with eGFR and lipid profile. IG count was positively associated with current smoking, Killip class, and triglyceride concentration and inversely associated with LVEF and HDL-cholesterol. In patients with ST-Segment Elevation Myocardial Infarction (STEMI), NE-WY and IG concentration were significantly higher than in patients with Non-ST Elevation Myocardial Infarction (NSTEMI), whereas there was no difference in MO-SSC between these two patient groups.

Overall, IG, together with MO-SSC and NE-WY, were the leukocyte parameters most strongly associated with CV mortality. ROC analysis showed an AUC of 0.709 for NE-WY (95% confidence interval: 0.617 to 0.801, p=0.0002; **Supplementary Figure 4A**), an AUC of 0.540 for MO-SSC (95% confidence interval: 0.439 to 0.641, p=0.469; **Supplementary Figure 4B**) and an AUC of 0.761 for IG (95% confidence interval: 0.680 to 0.843, p<0.0001; **Supplementary Figure 4C**). In comparison, parameters known to be strongly associated with mortality, such as neutrophil concentration, NLR, and hs-CRP, showed similar results (neutrophil concentration: AUC = 0.7038, 95% confidence interval: 0.615 to 0.793, p=0.0002; **Supplementary Figure 4D**; NLR: 0.7091, 95% confidence interval: 0.602 to 0.816, p=0.0001; **Supplementary Figure 4E**; hs-CRP: 0.7280, 95% confidence interval: 0.635 to 0.821, p<0.0001; **Supplementary Figure 4F**).

**Figure 2:**
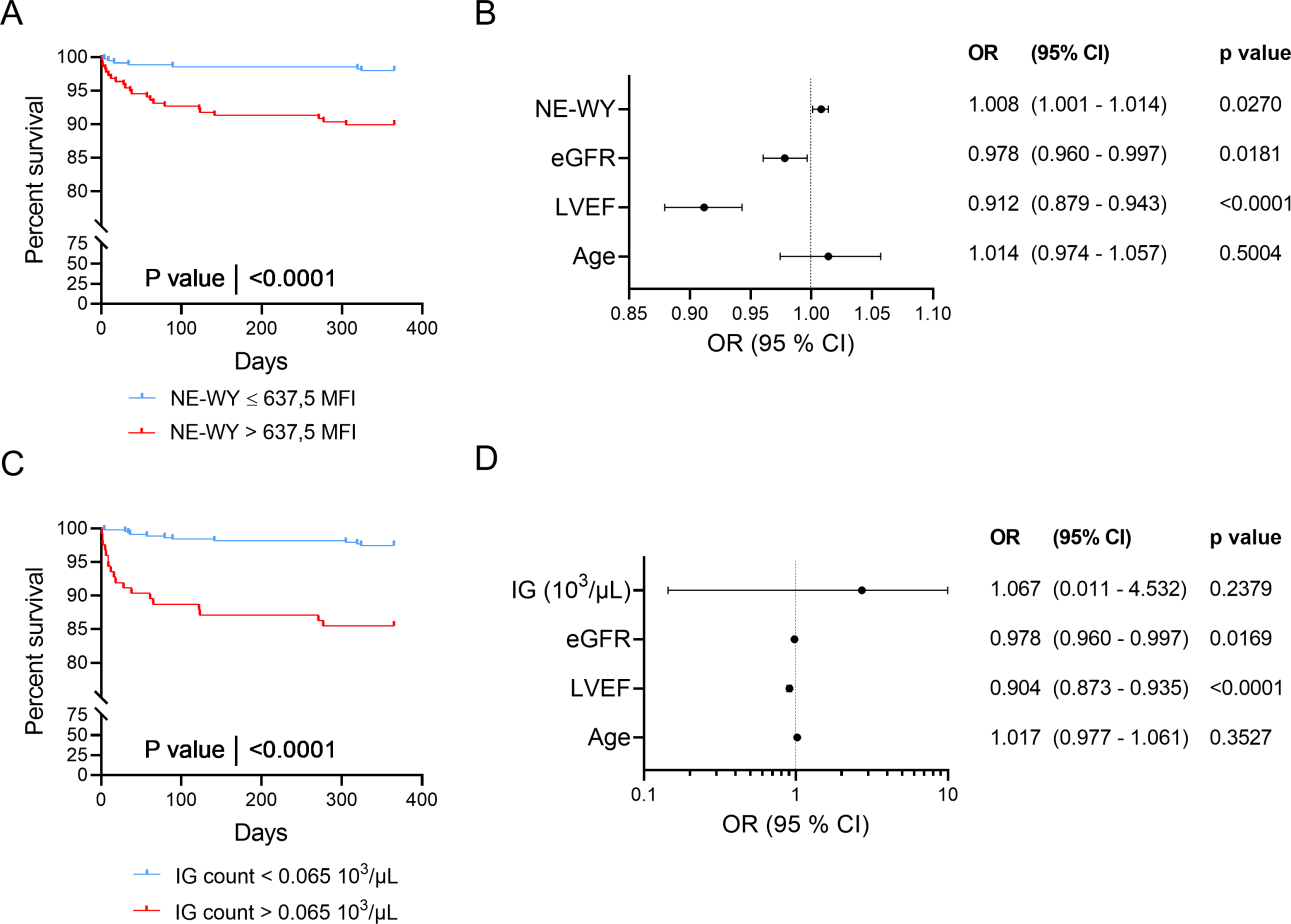
Predictive value of neutrophil heterogeneity in fluorescence intensity and immature granulocyte concentration for cardiovascular mortality. **(A, C)** Kaplan-Meier curve of CV mortality stratified by NE-WY **(A)** or IG count **(C)**. Patients were categorized into two groups based on NE-WY value or IG count. The optimal ratio for discriminating mortality was determined by using Youden’s Index from the ROC curve. Statistical significance was assessed using the log-rank test (n_patients_=558); **(B, D)** Forest plots showing Odd Ratio values and 95% CI of multivariate logistic regression analysis for NE-WY **(C)** or IG count **(F)**, adjusted for confounding factors, and CV mortality (n_patients_=558). OR, Odd Ratio; CI, confidence intervals; NE, Neutrophils; WY, heterogeneity in fluorescence intensity; IG, Immature Granulocytes; CV, cardiovascular.

Optimal thresholds, determined using the Youden index, were 637.5 MFI for NE-WY (sensitivity: 75.86%, specificity: 63.33%) and 0.065×10³/µL for IG (sensitivity: 62.07%, specificity: 79.96%) (**Supplementary Table 4**). These cut-offs were used to stratify patients into high- and low-risk groups. Kaplan–Meier analysis showed that patients with NE-WY and IG values above these thresholds had significantly lower survival rates, supporting their association with CV mortality (**Figures 2A, C**).

Finally, given the association between these leukocyte parameters and established predictors of CV mortality, particularly LVEF, multivariate logistic regression analyses were performed with four variables included per model (**Figures 2B, D**). NE-WY remained significantly associated with CV mortality after adjustment for age, LVEF, and eGFR. In contrast, IG concentration was no longer significant in the multivariate model. This analysis suggests that NE-WY is an interesting biomarker for assessing the risk of CV mortality.

### Inflammatory markers show distinct correlation patterns with cellular concentrations and CPD parameters

To better understand the relationship between leukocyte populations and inflammation, we compared cell counts with several systemic inflammatory markers, including IL-1β, IL-6, MPO, and hs-CRP. As previously reported, IL-1β and hs-CRP levels were higher in patients who died from CV causes (15). We observed the same pattern for IL-6 and MPO, which were also elevated in deceased patients compared to survivors (IL-6: 26.89 vs 6.15 pg/mL, p = 0.016; MPO: 64.17 vs 37.5 pg/mL, p = 0.0004; **Supplementary Figure 5**).

**Figure 3:**
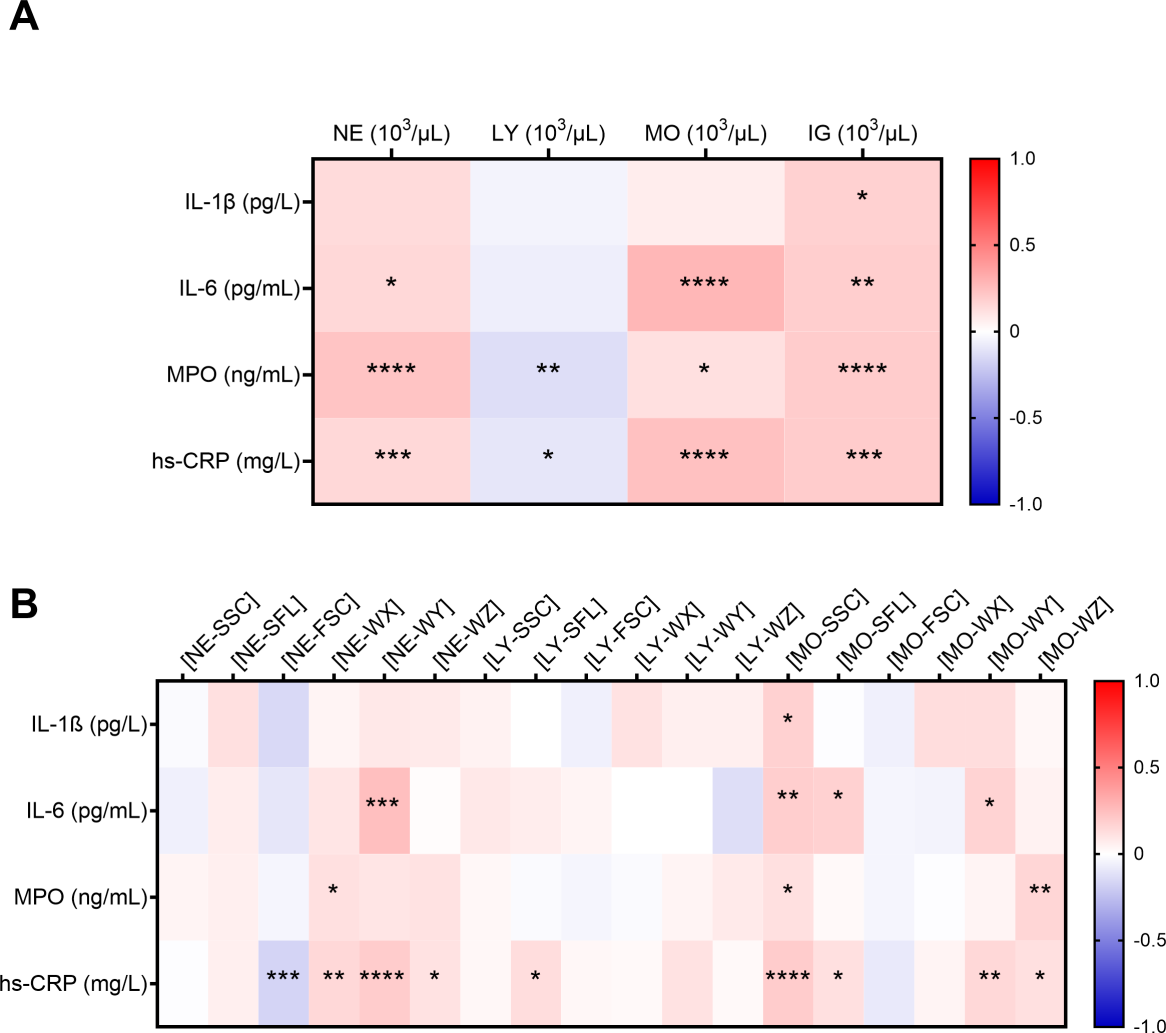
Inflammatory markers show distinct correlation patterns with cell population data. Heatmaps illustrating the Spearman correlation coefficients between IL-1β (n_patients_=217), IL-6 (n_patients_=301), MPO (n_patients_=496) or hs-CRP (n_patients_=572) and cell counts **(A)** or fluorescent parameters **(B)**. Each cell represents the correlation coefficient, with values ranging from -1 (blue) to +1 (red). Spearman test was performed for all tests. Significance was assessed using the Benjamini-Hochberg correction for multiple comparisons. *p<0.05, **p<0.01, ***p<0.001, ****p<0.0001. IL, interleukin; MPO, myeloperoxidase; hs-CRP, high sensitive C reactive protein; NE, Neutrophils; LY, Lymphocytes; MO, Monocytes; IG, Immature Granulocytes; SSC, side scatter; SFL, side fluorescence light intensity; FSC, forward scatter; WX, heterogeneity in cell complexity; WY, heterogeneity in fluorescence intensity; WZ, heterogeneity in size.

Correlation analyses between the four inflammatory markers and cell counts (**Figure 3A**) showed that IL-1β was positively associated only with IGs (r = 0.18, p = 0.030). In contrast, IL-6, MPO, and hs-CRP were all significantly and positively correlated with neutrophil, monocyte, and IG counts. Lymphocytes tended to decrease with higher inflammatory markers, and this inverse relationship was significant for MPO and hs-CRP.

We then analysed the same inflammatory markers in relation to CPD parameters (**Figure 3B**). As before, IL-1β showed almost no associations, except with MO-SSC (r = 0.18, p = 0.030). IL-6 correlated only with NE-WY (r = 0.25, p < 0.0001) among neutrophil parameters, but showed several associations with monocyte parameters (MO-SSC, MO-SFL, MO-WY). MPO showed relatively few correlations with CPD, limited to NE-WX, MO-SSC, and MO-WZ. In contrast, hs-CRP displayed numerous significant associations: it was negatively correlated with neutrophil size (NE-FSC) but positively correlated with their heterogeneity parameters (NE-WX, NE-WY, NE-WZ). For monocytes, hs-CRP was associated with increased complexity and fluorescence (MO-SSC, MO-SFL), as well as greater heterogeneity (MO-WY, MO-WZ). For lymphocytes, the only significant association was between hs-CRP and LY-SFL.

Overall, these results confirm that neutrophils, monocytes, and IG are the cell populations most strongly linked to elevated inflammatory markers. Among CPD, NE-WY, and MO-SSC seem to be the most closely and positively associated with systemic inflammation.

### Associations between fatty acid profiles and neutrophil activation markers

After examining the relationships between cellular and protein markers, we assessed how leukocyte counts and CPD parameters were related to plasma FA. Notably, MO-SSC and IG count were inversely associated with several lipid profile markers, particularly HDL-cholesterol, suggesting a potential interplay between monocyte activation, myelopoiesis, and lipid metabolism.

**Figure 4:**
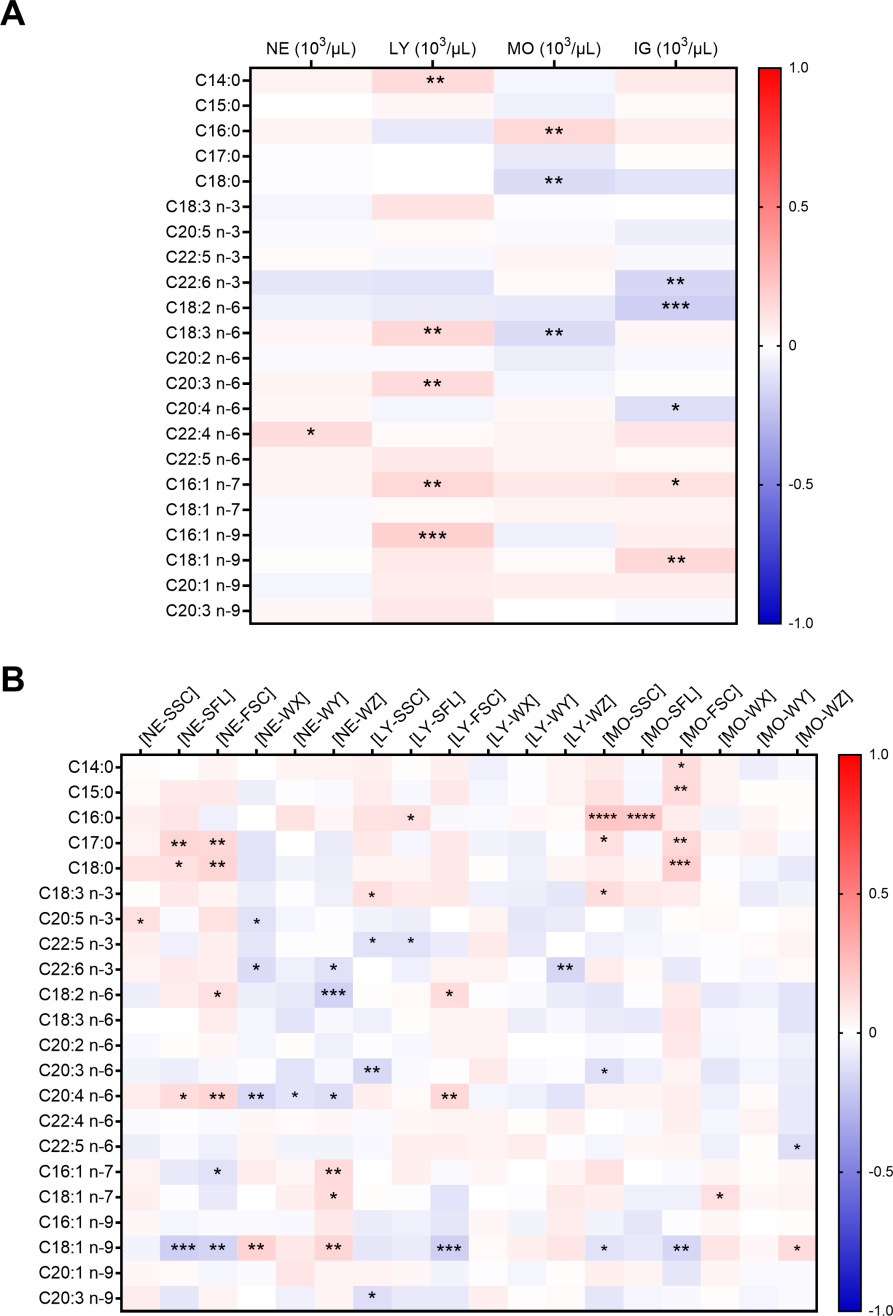
Main associations between plasma fatty acids and cell population data. Heatmaps illustrating the Spearman correlation coefficients between plasma fatty acid and cell counts **(A)** or fluorescent parameters **(B)** (n_patients_=572). Each cell represents the correlation coefficient, with values ranging from -1 (blue) to +1 (red). Spearman test was performed for all tests. Significance was assessed using the Benjamini-Hochberg correction for multiple comparisons. *p<0.05, **p<0.01, ***p<0.001, ****p<0.0001. NE, Neutrophils; LY, Lymphocytes; MO, Monocytes; IG, Immature Granulocytes; SSC, side scatter; SFL, side fluorescence light intensity; FSC, forward scatter; WX, heterogeneity in cell complexity; WY, heterogeneity in fluorescence intensity; WZ, heterogeneity in size.

For leukocyte counts (**Figure 4A**), neutrophils showed very limited relationships with fatty acids. In contrast, lymphocytes and monocytes were linked to several FA. Lymphocyte counts were mainly positively correlated with saturated and monounsaturated FA. Monocytes showed more variable relationships, with positive correlations for some FA (e.g., palmitic acid) and negative correlations for others (e.g., stearic and γ-linolenic acids).

IG displayed a distinct profile, characterized by positive correlations with monounsaturated FA (e.g., C16:1 n-7, C18:1 n-9) and negative correlations with several polyunsaturated FA (e.g., C22:6 n-3, C18:2 n-6, C20:4 n-6).

For CPD (**Figure 4B**), clearer patterns emerged. Saturated FA were mainly related to neutrophil and monocyte activation markers, particularly cell complexity and fluorescence. Palmitic acid showed strong relationships, notably with monocyte complexity and fluorescence (MO-SSC, MO-SFL), while other SFA were mainly related to cell size.

Monounsaturated FA showed a mixed profile, with inverse correlations with activation markers but positive correlations with heterogeneity indices, suggesting more complex effects on leukocyte activation.

Polyunsaturated FA showed more heterogeneous relationships. Omega-3 FA were only weakly related to leukocyte parameters, whereas omega-6 FA were linked to both cell size and activation markers, particularly in neutrophils and lymphocytes.

Overall, these analyses show specific, FA–dependent associations with leukocyte counts and activation markers (CPD), highlighting distinct patterns for saturated, monounsaturated, and PUFA.

## Discussion

From this contemporary cohort of MI patients, we present comprehensive and consistent data on the association between leukocyte CPD, systemic inflammatory markers, and mid-term CV prognosis, and we open the discussion on potential pathophysiological links. While CPD offers additional information beyond routine blood counts, its prognostic performance appears comparable to that of conventional inflammatory markers and neutrophil counts. Nevertheless, our findings support their potential value as accessible biomarkers reflecting immune activation in clinical practice.

AMI triggers a tightly regulated inflammatory response that plays a central role in both tissue injury and repair. Following ischemic necrosis, damage-associated molecular patterns (DAMPs) activate innate immune pathways, leading to rapid recruitment of neutrophils and monocytes to the injured myocardium. Neutrophils are among the first responders, contributing to debris clearance but also to collateral tissue damage through the release of proteolytic enzymes, reactive oxygen species, and neutrophil extracellular traps. This initial phase is followed by monocyte/macrophage infiltration, which supports phagocytosis and orchestrates tissue remodeling. While this inflammatory cascade is essential for healing, its excessive or prolonged activation can exacerbate myocardial injury, impair ventricular function, and promote adverse remodeling, ultimately increasing the risk of heart failure and cardiovascular mortality. Circulating inflammatory mediators such as IL-1β, IL-6, and hs-CRP reflect the magnitude of this response and have been consistently associated with clinical outcomes, highlighting inflammation as both a marker and a driver of AMI prognosis (19).

Our results highlight a consistent relationship between myocardial injury and neutrophil activation. TnI AUC was positively correlated with neutrophil count and multiple CPD parameters, including SSC, SFL, FSC, and NE-WY, indicating that greater myocardial damage is associated not only with increased neutrophil levels but also with changes in cell morphology and activation states. This relationship is further supported by the comparison between surviving patients and those who experienced first-year CV death, in which higher neutrophil concentrations and NE-WY fluorescence intensity (a marker of neutrophil activation and heterogeneity) were associated with increased CV mortality. The increased NE-WY values likely reflect enhanced neutrophil degranulation, oxidative burst, or metabolic reprogramming, processes known to contribute to tissue damage, sustained inflammation, and adverse cardiovascular outcomes (20). This aligns with existing evidence that neutrophil infiltration and activation are key components of the inflammatory response following cardiac injury, potentially contributing to tissue damage and contributing to adverse outcomes (3, 21, 22).

The particularly strong positive correlations between IG concentration and both TnI AUC and first-year mortality, through reduced LVEF, highlight the potential role of emergency granulopoiesis in response to myocardial stress and neutrophil involvement in myocardial damage (13). Moreover, NE-WY and IG concentration are significantly elevated in STEMI patients, suggesting that coronary occlusion may enhance neutrophil recruitment and their inflammatory response.

The negative correlation between TnI AUC and lymphocyte SFL, alongside the observation of lower lymphocyte counts in non-survivors, is consistent with other studies showing an unfavorable CV prognosis in patients with lymphopenia (23, 24). Our observations are further corroborated by recent studies demonstrating that elevated NLR is a robust biomarker of acute myocardial injury and adverse clinical outcomes in CV patients (5, 6).

Monocyte-related findings were less consistent. Although higher monocyte counts were observed in non-survivors, CPD parameters showed limited changes. The heterogeneous associations observed with TnI AUC may reflect the coexistence of different monocyte subsets with distinct functional roles, which cannot be discriminated using standard CPD measurements (25).

Among all leukocyte-related parameters, the three markers associated with mortality and myocardial necrosis—beyond neutrophil and monocyte count—were IG concentration, MO-SSC and NE-WY. IG concentration has already been described as a predictive marker for the occurrence of major cardiovascular events (12, 13). Although MO-SSC is statistically associated with cardiovascular mortality, the ROC curve shows an AUC of 0.54, which is insufficient for a decision-making marker. Conversely, relatively little data exist on the link between NE-WY and CV damage. This parameter represents the heterogeneity in neutrophil fluorescence intensity, reflecting the diversity within the neutrophil population regarding their nucleic acid content, which is affected by cell activation, maturity, and cellular death processes. It has already been documented in various infectious diseases, either as a predictive marker of sepsis or mortality in the case of certain viral hemorrhagic fevers (26, 27). Moreover, elevated preoperative neutrophil granularity and fluorescence heterogeneity correlate with higher risks of postoperative complications such as, renal failure, supraventricular arrhythmia, stroke, and mortality in cardiac surgery patients (10). Our data show that, beyond the correlation between NE-WY and TnIc AUC, NE-WY was inversely related to LVEF and renal function, and positively associated with diabetes and hs-CRP, supporting its link with both organ dysfunction, metabolic disorders and systemic inflammation. Diabetes is known to induce a pro-inflammatory environment (28), and the elevated NE-WY values in diabetic patients may reflect enhanced neutrophil activation, which could exacerbate vascular damage and contribute to the increased CV risk observed in this population. Then, among all medications taken by patients, only aspirin could impact the immune response, since patients taking other drugs that affect the immune system were not included in the cohort. However, this one has been used in low doses as an antiplatelet therapy, which explains the lack of significant association with CPD.

Interestingly, classical lipid parameters, including total cholesterol, LDL-C, HDL-C, triglycerides, and Lp(a), did not show significant associations with NE-WY intensity, suggesting that neutrophil activation in this context may be more directly influenced by acute inflammatory or metabolic stressors rather than traditional lipid-related pathways. Therefore, NE-WY emerges as an interesting indicator of major adverse cardiovascular events, a finding further supported by our multivariate logistic regression model that incorporates the principal parameters associated with cardiovascular mortality.

To explore further pathophysiological components linking CPD, inflammation and major adverse cardiovascular events, we also analyzed the profile of inflammatory markers, such as IL-1β, IL-6, MPO and hs-CRP. Our previous results showed that detectable levels of IL-1β, as well as high levels of hs-CRP, were associated with a significant increase in CV mortality (15), consistent with the literature (29). The same observations apply here to IL-6 and MPO, which were higher in patients who died from CV causes. When we compared the concentration of these inflammatory parameters with the cell count, we observed that neutrophils and monocytes were mainly positively associated with the inflammation, while lymphocytes were inversely correlated, particularly with hs-CRP. Among all cellular populations, only IG showed a significant correlation with IL-1β, which is known to enhance hematopoiesis in murine models (30). This finding supports an additional explanation for the deleterious effects of IL-1β through the recruitment of new, potentially harmful granulocyte progenitors. Concerning CPD, the two main parameters significantly associated with these inflammatory parameters are the neutrophil heterogeneity of fluorescence and monocyte complexity, which is also consistent with our findings on mortality. Notably, the positive correlations between IL-6 and neutrophil-related parameters, such as neutrophil concentration and heterogeneity of fluorescence and IG concentration, suggest that IL-6 not only reflects the magnitude of inflammation but actively drives neutrophil mobilization and activation. This aligns with previous findings demonstrating that elevated IL-6 levels are associated with increased neutrophil counts, which in turn contribute to tissue damage and adverse CV outcomes (31, 32). Furthermore, associations between neutrophils and MPO further reinforce the link between systemic inflammation and neutrophil-mediated tissue damage. MPO, an enzyme specifically released by neutrophils (33), and in a lesser proportion by monocytes (34), is positively correlated with neutrophil and IG concentration, suggesting that MPO serves as a downstream effector of IL-6-driven neutrophil activation.

Finally, the present study highlights previously unrecognized associations between circulating FA profiles and leukocyte activation markers assessed through CPD, suggesting that specific lipid environments may shape innate immune responses in the acute phase of MI (8). SFA, particularly palmitic acid, were consistently linked to increased monocyte and neutrophil activation, in line with experimental evidence showing that SFA can trigger inflammatory pathways and promote myeloid cell priming (35). The inverse associations observed between IGs and several PUFA, including DHA, arachidonic acid, and linolenic acid, support the anti-inflammatory role of PUFA and suggest that lower PUFA availability may contribute to enhanced emergency granulopoiesis in AMI patients (9, 36). On the other hand, MUFA and PUFA seem to have a complex inverse effect on neutrophils. Indeed, neutrophil scatter and fluorescence are positively correlated with PUFA and inversely correlated with MUFA, whereas their heterogeneity parameters show the exact opposite associations, underscoring the nuanced interplay between fatty acid composition and neutrophil activation states.

Together, these findings indicate that the balance between SFA, MUFA, and PUFA may modulate the inflammatory profile captured by CPD, potentially influencing downstream cardiovascular outcomes. Although causal mechanisms cannot be inferred from this observational dataset, our results raise the possibility that FA composition may modulate neutrophil activation and myeloid response after AMI, warranting further mechanistic studies and prospective validations.

The main limitation of this study is that it is an observational and single-center, limiting causal interpretation and external generalizability. Thus, the results should be interpreted with caution. In addition, the relatively low number of cardiovascular deaths may limit statistical power and the precision of risk estimates and raise the possibility of overfitting in multivariable analyses. Although we report significant associations between CPD parameters, inflammatory markers, and cardiovascular outcomes, our findings should be considered as preliminary and require confirmation in independent, larger, and multicenter cohorts to ensure their robustness and generalizability across diverse populations with different clinical practices and hematologic analyzers. An interesting approach would be to perform a CPD monitoring following the acute ischemic event to observe their changes in conjunction with inflammation. Moreover, it would be interesting to perform functional tests to determine if there is a specific response of neutrophil during MI, such as NET formation, and whether treatment with certain FA could modulate this response.

## Data Availability

The datasets presented in this article are not readily available because indirect nominative data cannot be shared publicly under French laws, we cannot upload our minimal underlying data ser, the datasets generated and/or analyzed during the current study are not publicly available but are available from the corresponding author on reasonable request. Requests to access the datasets should be directed to Damien LELEU, damien.leleu@ube.fr.
