# Supplementary material for: Leukocyte activation and inflammation in acute myocardial infarction: novel insights from cell population data
Source: Front Immunol. 2026 Jul 3;17:1864687. doi: 10.3389/fimmu.2026.1864687 (PMC13375499; doi:10.3389/fimmu.2026.1864687)
Supplement: Supplementary file 1 [file DataSheet1.docx]

|  | | **All patients**  **(N=572)** | **Living**  **(N=529)** | **CV Death**  **(N=29)** | ***P* value** |
| --- | --- | --- | --- | --- | --- |
| **Risk factors** | |  |  |  |  |
|  | Age. *y* | 68 [58; 77] | 67 [57; 76] | 76 [67; 86] | 0.0017 |
|  | Female | 164 (28.6%) | 154 (28.8%) | 8 (27.6%) | 1.0000 |
|  | Diabetes | 137 (23.9%) | 121 (22.7%) | 11 (37.9%) | 0.0732 |
|  | Current smoking | 177 (31.72%) | 170 (32.14%) | 7 (24.14%) | 0.4194 |
|  | BMI. kg/m² | 27 [24; 30] | 27 [24; 30] | 24 [23; 29] | 0.0096 |
| **Clinical parameters** | |  |  |  |  |
| STEMI | | 306 (53.49%) | 283 (53.5%) | 19 (65.52%) | 0.2521 |
|  | CAD | 118 (20.6%) | 102 (19.1%) | 11 (37.9%) | 0.0289 |
|  | Anterior wall infarction | 133 (23.25%) | 119 (22.50%) | 12 (41.38%) | 0.0251 |
|  | LVEF (%) | 55 [45; 60] | 55 [45; 60] | 34 [26.5; 50.5] | <0.0001 |
|  | eGFR (CKD-EPI. mL/min/1.73m²) | 88 [68; 98] | 89 [71; 99] | 58 [39; 90] | 0.0003 |
|  | GRACE score | 139 [117; 163] | 137 [115; 161] | 183 [165; 207.5] | <0.0001 |
|  | Killip class ≥ 3 | 21 (3.67%) | 14 (2.65%) | 6 (20.69%) | 0.0003 |
| **Biological data** | |  |  |  |  |
|  | Total cholesterol (mmol/L) | 5.22 [4.38; 5.99] | 5.29 [4.50; 6.07] | 3.78 [3.16; 4.90] | <0.0001 |
|  | LDL cholesterol (mmol/L) | 3.37 [2.48; 4.06] | 3.45 [2.59; 4.08] | 2.49 [1.48; 3.25] | <0.0001 |
|  | HDL cholesterol (mmol/L) | 1.14 [0.93; 1.37] | 1.15 [0.93; 1.38] | 0.95 [0.74; 1.34] | 0.0157 |
|  | Triglycerides (mmol/L) | 1.35 [0.93; 18.18] | 1.36 [0.94; 18.18] | 1.08 [0.79; 1.71] | 0.1206 |
|  | Lp(a) (mg/L) | 179.0 [100.0; 538.5] | 182.0 [100.0; 543.5] | 100.0 [100.0; 415.0] | 0.1973 |
|  | HbA1c (%) | 5.80 [5.60; 6.40] | 5.80 [5.60; 6.30] | 6.10 [5.80; 7.30] | 0.0532 |
|  | Troponin Ic pic (ng/mL) | 14947 [1893; 67649.5] | 13431 [1771; 64587] | 54817.5 [6457.25; 99506] | 0.0159 |
|  | Troponin Ic AUC (ng/mL/h) | 5.85e+05 [1.11e+05; 2.29e+06] | 5.78e+05 [1.07e+05; 2.12e+06] | 2.56e+06 [2.60e+05; 4.95e+06] | 0.0016 |
|  | BNP (pg/mL) | 412 [115.5; 1615] | 359.5 [108; 1390.3] | 5186 [580; 10143] | <0.0001 |
|  | CRP (mg/L) | 3.71 [1.29; 11.30] | 3.44 [1.24; 10.00] | 11.40 [4.05; 67.60] | <0.0001 |
|  | IL-6 (pg/mL) | 6.44 [3.46; 21.67] | 6.15 [3.38; 18.66] | 26.89 [6.36; 37.31] | 0.0172 |
|  | MPO (ng/mL) | 38.51 [25.84; 59.77] | 37.50 [25.72; 58.08] | 64.17 [39.34; 115.83] | 0.0005 |
|  | IL-1β > 0.05 pg/mL | 216 (37.7%) | 191 (35.8%) | 18 (62.1%) | 0.0092 |
| **Treatment** | |  |  |  |  |
|  | Aspirin | 137 (23.9%) | 120 (22.5%) | 10 (34.5%) | 0.1737 |
|  | Statin | 172 (30.0%) | 158 (29.6%) | 10 (34.5%) | 0.6778 |

**Table S1**: Descriptive parameters of all, living, and cardiovascular deceased patients (n (%) or median (IQR)).

Mann Whitney and Spearman tests were performed. *BMI: Body mass index; STEMI: ST-Segment Elevation Myocardial Infarction; CAD: coronary artery disease; LVEF: left ventricular ejection fraction; eGFR: estimation of Glomerular filtration rate; GRACE: Global Registry of Acute Coronary Events; eGFR: estimated glomerular filtration rate; LDL: Low density lipoprotein; HDL: high density lipoprotein; Lp(a): Lipoprotein(a); HbA1c: Haemoglobin A1c; AUC: Area under the curve; BNP: Brain natriuretic peptide; CRP: C-reactive protein; IL: interleukin; MPO: myeloperoxidase*

**Table S2: NE-WY according to clinical and biological characteristics.**

|  |  | | **Mean (± SD)**  **or n (%)** | **r**  **or NE-WY median [IQR]** | **p-value** |
| --- | --- | --- | --- | --- | --- |
|  | Age | | 67.5 (± 13.17) | 0.1624 | <0.0001 |
|  | Female | no | 408 (71.32%) | 626.5 [606.0-653.0] | 0. 7479 |
|  |  | Yes | 164 (28.68%) | 629.5 [605.3-650.9] |  |
|  | BMI, kg/m² | | 27.2 (± 4.768) | -0.0122 | 0.7714 |
|  | Diabetes | no | 435 (76.05%) | 623.0 [602.0-650.0] | 0.0003 |
|  |  | Yes | 137 (23.95%) | 639.0 [617.5-659.5] |  |
|  | Current smoking | no | 389 (68.72%) | 629 [606-652] | 0.7208 |
|  |  | Yes | 177 (31.72%) | 626 [604-651] |  |
|  | CAD | no | 454 (79.37%) | 628.0 [606.0-653.3] | 0.5442 |
|  |  | Yes | 118 (20.63%) | 626.0 [602.8-648.0] |  |
|  | Killip class $\geq$ 3 | no | 545 (96.29%) | 626.0 [604.0-650.5] | <0.0001 |
|  |  | Yes | 21 (3.71%) | 676.0 [638.5-702.5] |  |
|  | Infract location | Anterior wall | 133 (49.81%) | 631.0 [611.0-656.0] | 0.9022 |
|  |  | Other Wall | 134 (50.19%) | 632.0 [611.0-655.0] |  |
|  | STEMI | no | 266 (46.51%) | 620 [599-645] | <0.0001 |
|  |  | Yes | 306 (53.49%) | 632.5 [611.25-659] |  |
|  | Aspirin treatment | no | 435 (76.05%) | 626.0 [604.0-651.0] | 0.1028 |
|  |  | Yes | 137 (23.95%) | 631.0 [610.0-661.0] |  |
|  | Statin treatment | no | 400 (69.93%) | 624.0 [604.0-650.0] | 0.0685 |
|  |  | Yes | 172 (30.07%) | 633.0 [610.0-658.0] |  |
|  | LVEF (%) | | 51.5 (± 10.68) | -0.1941 | <0.0001 |
|  | eGFR (mL/min/1.73 m²) | | 82.3 (± 24.04) | -0.1763 | <0.0001 |
|  | Total Cholesterol (mmol/L) | | 5.191 (± 1.29) | -0.0508 | 0.2258 |
|  | LDL-C (mmol/L) | | 3.325 (± 1.124) | -0.0823 | 0.0508 |
|  | HDL-C (mmol/L) | | 1.175 (± 0.3606) | -0.0303 | 0.4571 |
|  | Triglycerides (mmol/L) | | 1.662 (± 1.317) | 0.0519 | 0.2161 |
|  | Lp(a) (mg/L) | | 484.1 (± 423.6) | -0.0427 | 0.3896 |
|  | HbA1c (%) | | 6.21 (± 1.11) | 0.1067 | 0.0112 |

Mann Whitney and Spearman tests were performed. *SD: standard deviation; IQR: interquartile range; BMI: Body Mass Index; CAD: Coronary Artery Disease; STEMI: ST-Segment Elevation Myocardial Infarction; LVEF: Left Ventricular Ejection Fraction; eGFR: Estimated Glomerular Filtration Rate; AUC: Area Under the Curve); LDL-C: Low-Density Lipoprotein – Cholesterol; HDL: High-Density Lipoprotein – Cholesterol; Lp(a): Lipoprotein (a); HbA1c : Haemoglobin A1c.*

**Table S3: MO-SSC according to clinical and biological characteristics.**

|  |  | | **Mean (± SD)**  **or n (%)** | **r**  **or MO-SSC median [IQR]** | **p-value** |
| --- | --- | --- | --- | --- | --- |
|  | Age | | 67.5 (± 13.17) | 0.1316 | 0.0016 |
|  | Female | no | 408 (71.32%) | 119.6 [118.1–121.6] | 0.3295 |
|  |  | Yes | 164 (28.68%) | 120.1 [118.1–121.7] |  |
|  | BMI. kg/m² | | 27.2 (± 4.768) | 0.05839 | 0.1631 |
|  | Diabetes | no | 435 (76.05%) | 119.6 [117.9–121.5] | 0.0045 |
|  |  | Yes | 137 (23.95%) | 120.4 [118.8–122.1] |  |
|  | Current smoking | no | 389 (68.72%) | 119.8 [118.3-121.7] | 0.1700 |
|  |  | Yes | 177 (31.72%) | 119.8 [117.8-121.2] |  |
|  | CAD | no | 454 (79.37%) | 119.6 [117.8–121.4] | 0.0192 |
|  |  | Yes | 118 (20.63%) | 120.2 [118.7–122.2] |  |
|  | Killip class $\geq$ 3 | no | 545 (96.29%) | 119.8 [118.1-121.6] | 0.5291 |
|  |  | Yes | 21 (3.71%) | 120.2 [118.3-122.4] |  |
|  | Infract location | Anterior wall | 133 (49.81%) | 119.2 [117.7-121.4] | 0.9274 |
|  |  | Other Wall | 134 (50.19%) | 119.6 [117.6-121.3] |  |
|  | STEMI | no | 266 (46.51%) | 119.8 [118.4-121.7] | 0.52764 |
|  |  | Yes | 306 (53.49%) | 119.7 [118.0-121.5] |  |
|  | Aspirin treatment | no | 435 (76.05%) | 119.6 [117.9-121.5] | 0.0521 |
|  |  | Yes | 137 (23.95%) | 120.2 [118.5-121.9] |  |
|  | Statin treatment | no | 400 (69.93%) | 119.7 [118.0-121.6] | 0.8262 |
|  |  | Yes | 172 (30.07%) | 119.8 [118.3-121.5] |  |
|  | LVEF (%) | | 51.5 (± 10.68) | -0.07011 | 0.0959 |
|  | eGFR (mL/min/1.73 m²) | | 82.3 (± 24.04) | -0.1726 | <0.0001 |
|  | Total Cholesterol (mmol/L) | | 5.191 (± 1.29) | -0.1493 | 0.0003 |
|  | LDL-C (mmol/L) | | 3.325 (± 1.124) | -0.1624 | 0.0001 |
|  | HDL-C (mmol/L) | | 1.175 (± 0.3606) | -0.1624 | 0.0001 |
|  | Triglycerides (mmol/L) | | 1.662 (± 1.317) | -0.1386 | 0.0009 |
|  | Lp(a) (mg/L) | | 484.1 (± 423.6) | 0.09679 | 0.0208 |
|  | HbA1c (%) | | 6.21 (± 1.11) | 0.1389 | 0.0009 |

Mann Whitney and Spearman tests were performed. *SD: standard deviation; IQR: interquartile range; BMI: Body Mass Index; CAD: Coronary Artery Disease; STEMI: ST-Segment Elevation Myocardial Infarction; LVEF: Left Ventricular Ejection Fraction; eGFR: Estimated Glomerular Filtration Rate; AUC: Area Under the Curve); LDL-C: Low-Density Lipoprotein - Cholesterol, HDL: High-Density Lipoprotein – Cholesterol; Lp(a): Lipoprotein (a); HbA1c : Haemoglobin A1C.*

**Table S4: IG concentration according to clinical and biological characteristics.**

|  |  | | **Mean (± SD)**  **or n (%)** | **r**  **or IG median [IQR]** | **p-value** |
| --- | --- | --- | --- | --- | --- |
|  | Age | | 67.5 (± 13.17) | -0.022 | 0.6001 |
|  | Female | no | 408 (71.32%) | 0.04 [0.03- 0.06] | 0.8273 |
|  |  | Yes | 164 (28.68%) | 0.04 [0.02- 0.06] |  |
|  | BMI, kg/m² | | 27.2 (± 4.768) | 0.043 | 0.3033 |
|  | Diabetes | no | 435 (76.05%) | 0.04 [0.03- 0.06] | 0.8733 |
|  |  | Yes | 137 (23.95%) | 0.04 [0.02- 0.06] |  |
|  | Current smoking | no | 389 (68.72%) | 0,04 [0,02-0,06] | 0,0014 |
|  |  | Yes | 177 (31.72%) | 0,05 [0,03-0,07] |  |
|  | CAD | no | 454 (79.37%) | 0.04 [0.03- 0.07] | 0.2701 |
|  |  | Yes | 118 (20.63%) | 0.04 [0.03- 0.06] |  |
|  | Killip class $\geq$ 3 | no | 545 (96.29%) | 0.04 [0.02- 0.06] | <0.0001 |
|  |  | Yes | 21 (3.71%) | 0.08 [0.06- 0.17] |  |
|  | Infract location | Anterior wall | 133 (49.81%) | 0.04 [0.03- 0.07] | 0.7252 |
|  |  | Other Wall | 134 (50.19%) | 0.04 [0.02- 0.06] |  |
|  | STEMI | no | 266 (46.51%) | 0,03 [0,02-0,05] | <0.0001 |
|  |  | Yes | 306 (53.49%) | 0,04 [0,03-0,07] |  |
|  | Aspirin treatment | no | 435 (76.05%) | 0.04 [0.03- 0.06] | 0.5291 |
|  |  | Yes | 137 (23.95%) | 0.04 [0.02- 0.06] |  |
|  | Statin treatment | no | 400 (69.93%) | 0.04 [0.03- 0.06] | 0.0936 |
|  |  | Yes | 172 (30.07%) | 0.04 [0.02- 0.06] |  |
|  | LVEF (%) | | 51.5 (± 10.68) | -0.175 | <0.0001 |
|  | eGFR (mL/min/1.73 m²) | | 82.3 (± 24.04) | -0.044 | 0.2908 |
|  | Total Cholesterol (mmol/L) | | 5.191 (± 1.29) | 0.030 | 0.4749 |
|  | LDL-C (mmol/L) | | 3.325 (± 1.124) | 0.039 | 0.3572 |
|  | HDL-C (mmol/L) | | 1.175 (± 0.3606) | -0.168 | <0.0001 |
|  | Triglycerides (mmol/L) | | 1.662 (± 1.317) | 0.175 | <0.0001 |
|  | Lp(a) (mg/L) | | 484.1 (± 423.6) | -0.039 | 0.4368 |
|  | HbA1c (%) | | 6.21 (± 1.11) | 0.080 | 0.0570 |

Mann Whitney and Spearman tests were performed. *SD: standard deviation; IQR: interquartile range; BMI: Body Mass Index; CAD: Coronary Artery Disease; STEMI: ST-Segment Elevation Myocardial Infarction; LVEF: Left Ventricular Ejection Fraction; eGFR: Estimated Glomerular Filtration Rate; AUC: Area Under the Curve); LDL-C: Low-Density Lipoprotein - Cholesterol, HDL: High-Density Lipoprotein – Cholesterol; Lp(a): Lipoprotein (a); HbA1c : Haemoglobin A1C.*

**Table S5: Specificity, sensitivity, positive and negative predictive values of NE-WY and IG count threshold.**

|  | Threshold | Sp | Se | PPV | NPV |
| --- | --- | --- | --- | --- | --- |
| NE-WY | 637.5 | 63.33% | 75.86% | 10.19% | 97.95% |
| IG (10^3^/µL) | 0.065 | 79.96% | 62.07% | 14.52% | 97.47% |

The optimal threshold for discriminating CV mortality was determined using Youden’s index from the Receiver Operating Characteristic (ROC) curve. *NE-WY: neutrophil heterogeneity in fluorescence intensity; IG: immature granulocytes; Sp : specificity; Se: sensitivity; PPV: positive predictive value; NPV: negative predictive value.*


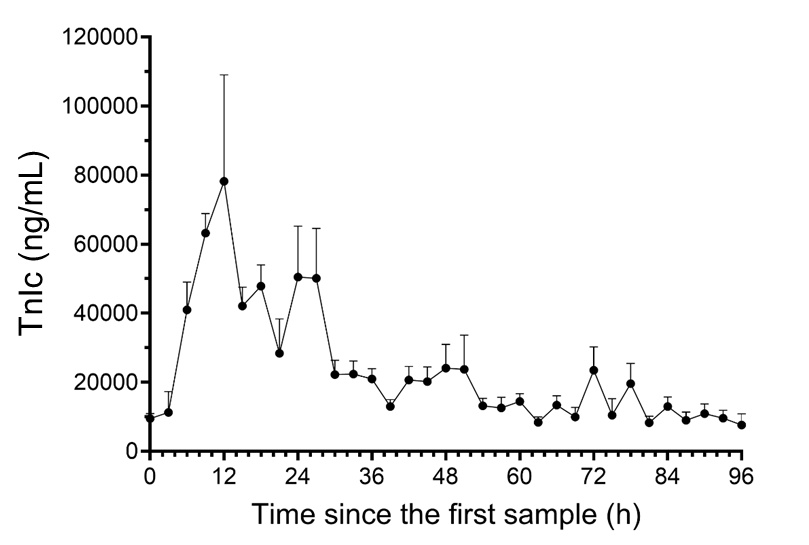


**Figure S1:** Average Troponin Ic concentration in the 96 hours following admission to intensive care. Means of 572 patients + SEM. *TnIc : Troponin Ic; H : Hours; SEM : standard error of the mean.*


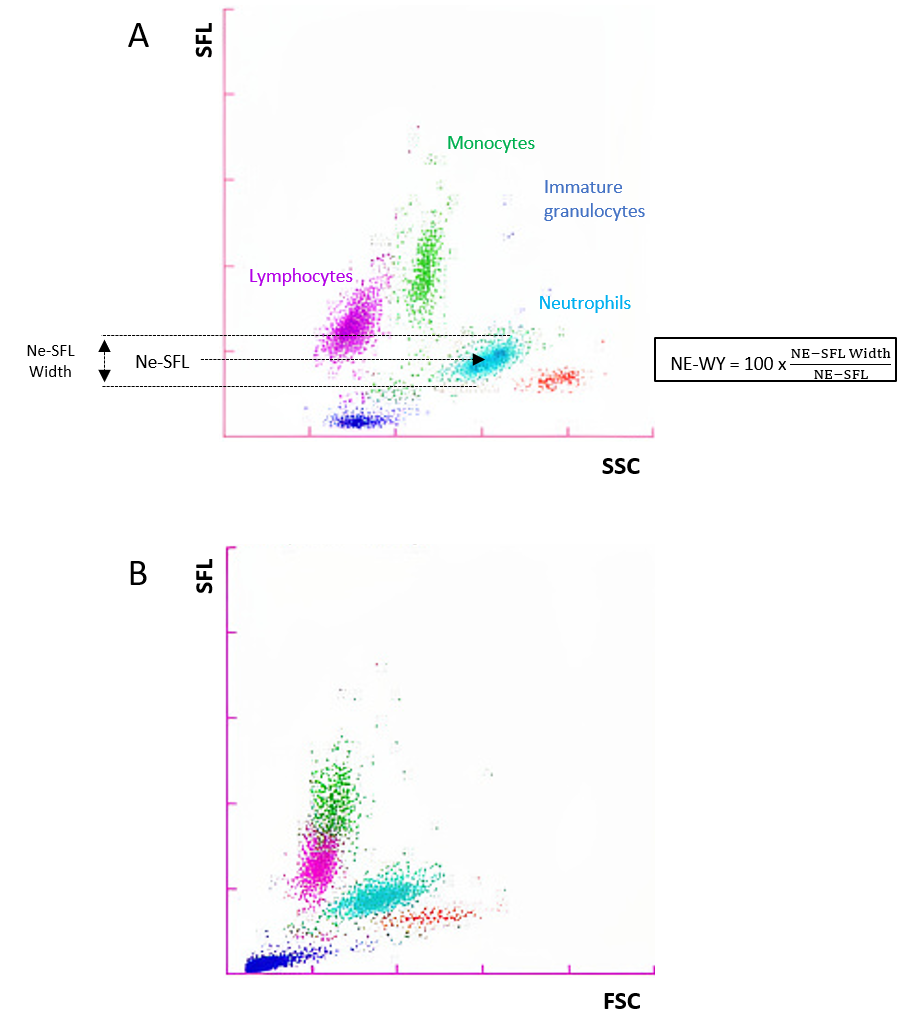


**Figure S2**: Scatter representation SFL vs SSC (A) and SFL vs FSC (B) of Cell Population Data.


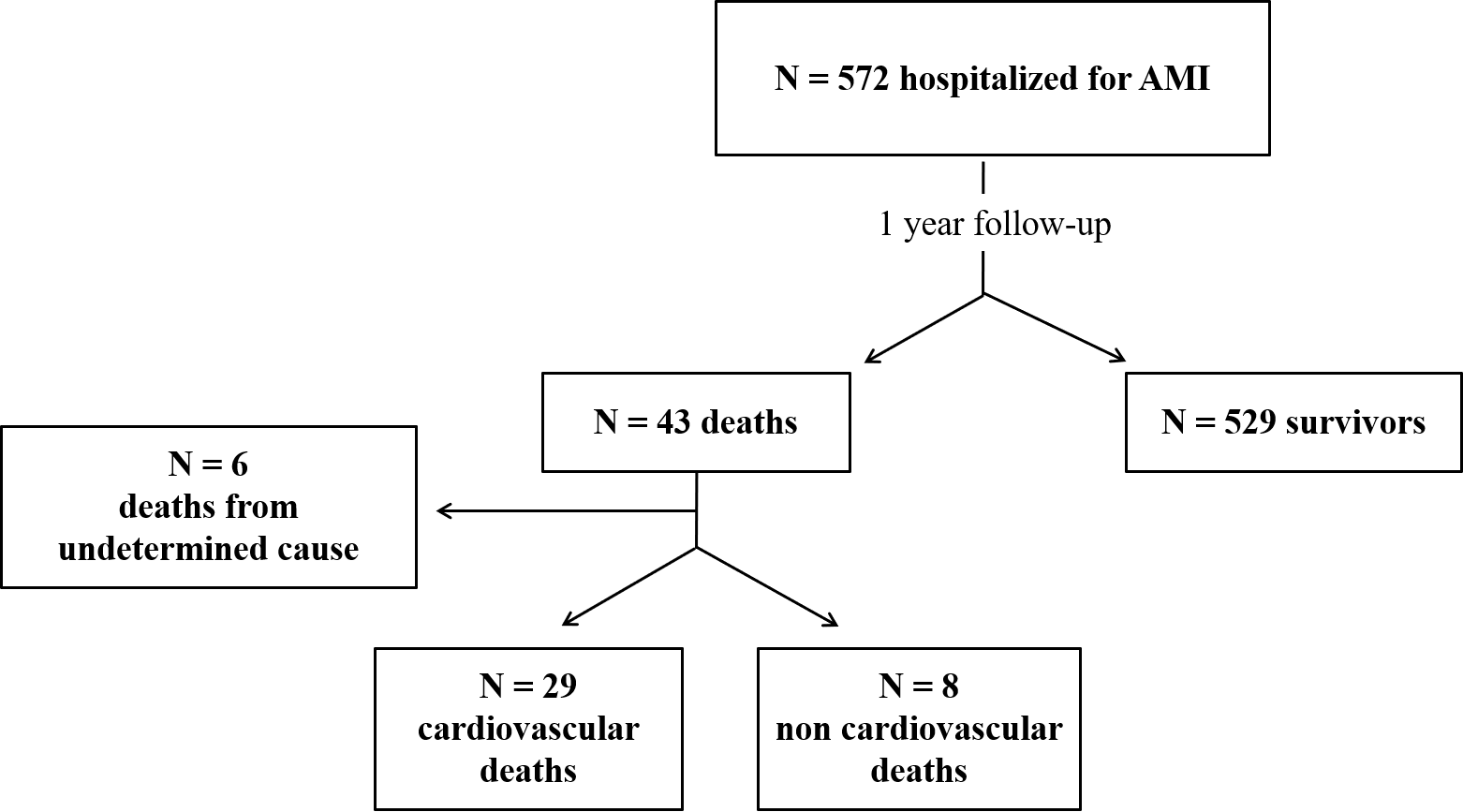


**Figure S3**: Flowchart of the study.


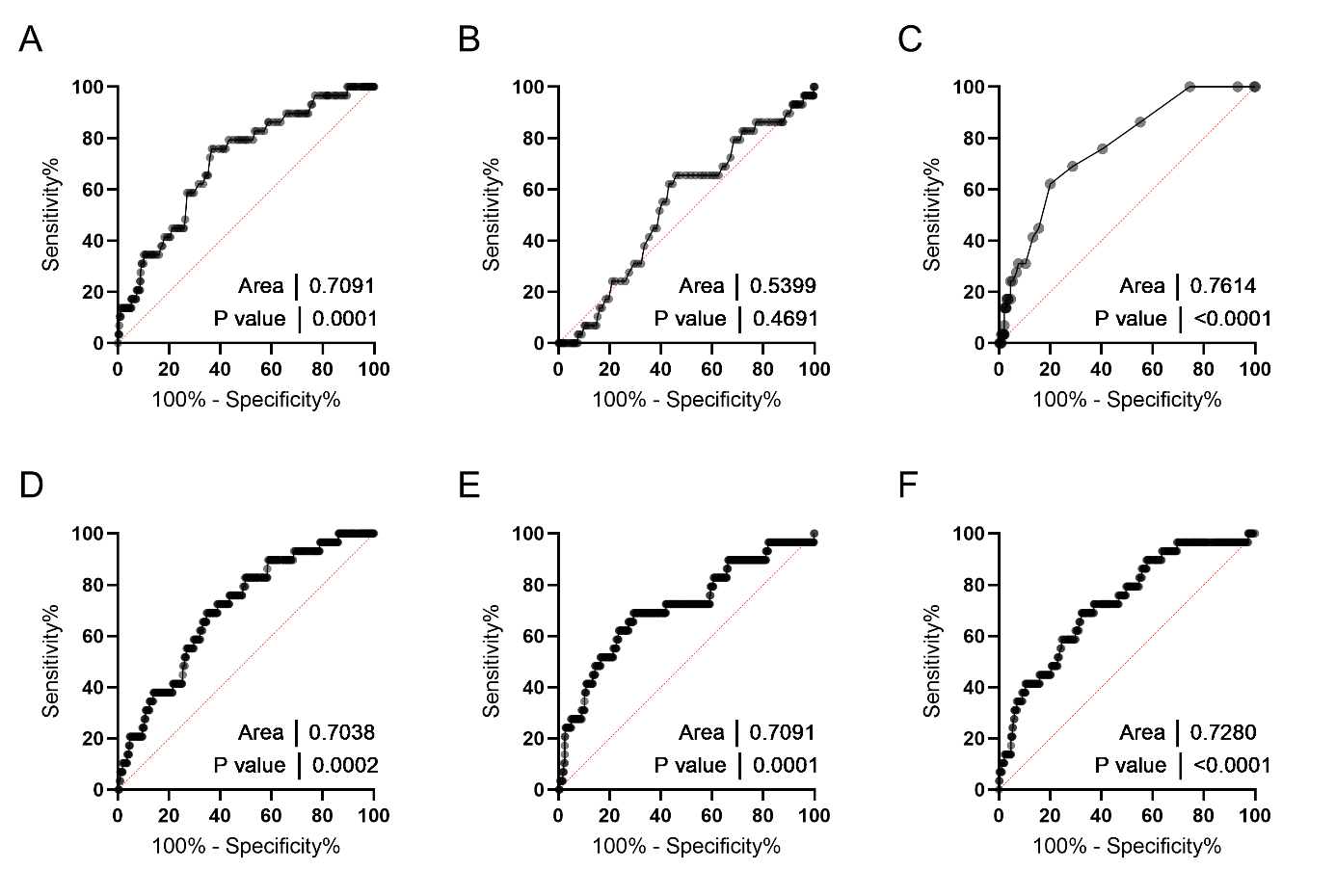


**Figure S4:** Receiver Operating Characteristic (ROC) curve showing the discriminative ability of NE-WY (A), MO-SSC (B), IG count (C), Neutrophil concentration (D), Neutrophil to Lymphocyte ratio (E) and hsCRP concentration (F) in predicting cardiovascular mortality (n_patients_=558).


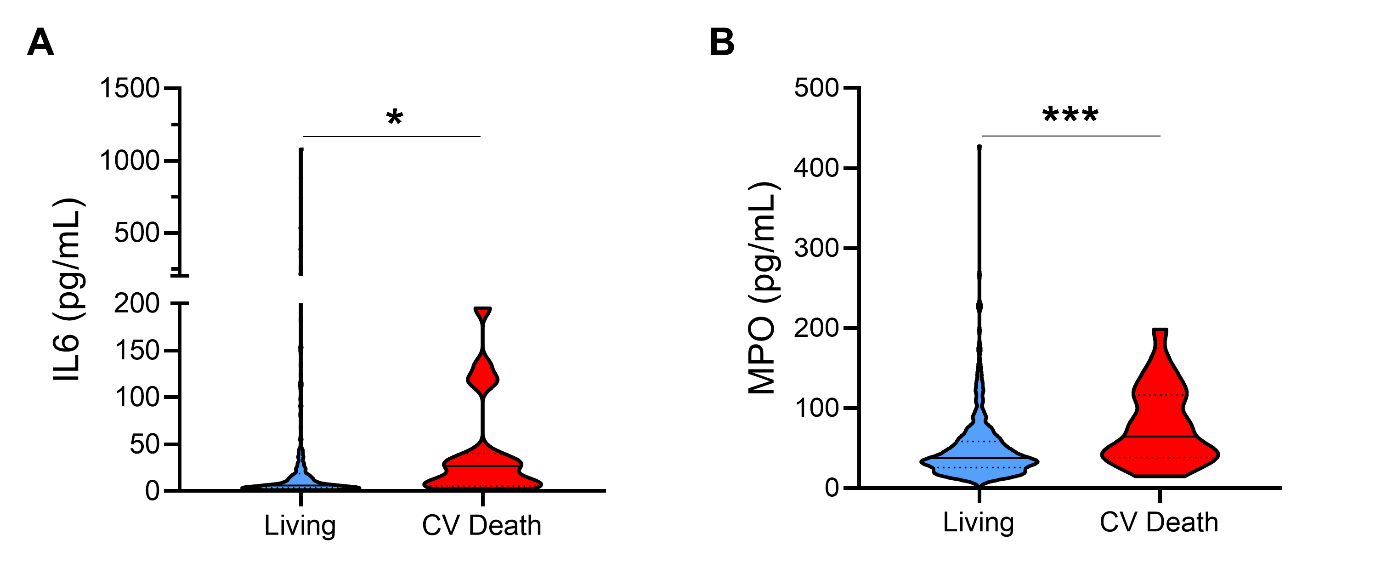


**Figure S5: IL-6 and MPO concentration according to cardiovascular death.** (A) Comparison of IL-6 concentrations between living patients and those deceased from cardiovascular causes, *: p<0.05, Mann-Whitney test; (B) Comparison of myeloperoxidase (MPO) concentrations between living patients and those deceased from cardiovascular causes, ***: p<0.001. Mann-Whitney test was performed.
